# Supplementary material for: Causal interplay between lactose intolerance and gut microbiota: a combined bidirectional Mendelian randomization and in vivo validation study
Source: Front Nutr. 2026 Jun 1;13:1803337. doi: 10.3389/fnut.2026.1803337 (PMC13265576; doi:10.3389/fnut.2026.1803337)
Supplement: Supplementary file 1 [file Data_Sheet_1.zip › supplementary materials/Forward/forest plot/ebi-a-GCST90027676.finngen_R12_E4_LACTONAS.pdf]

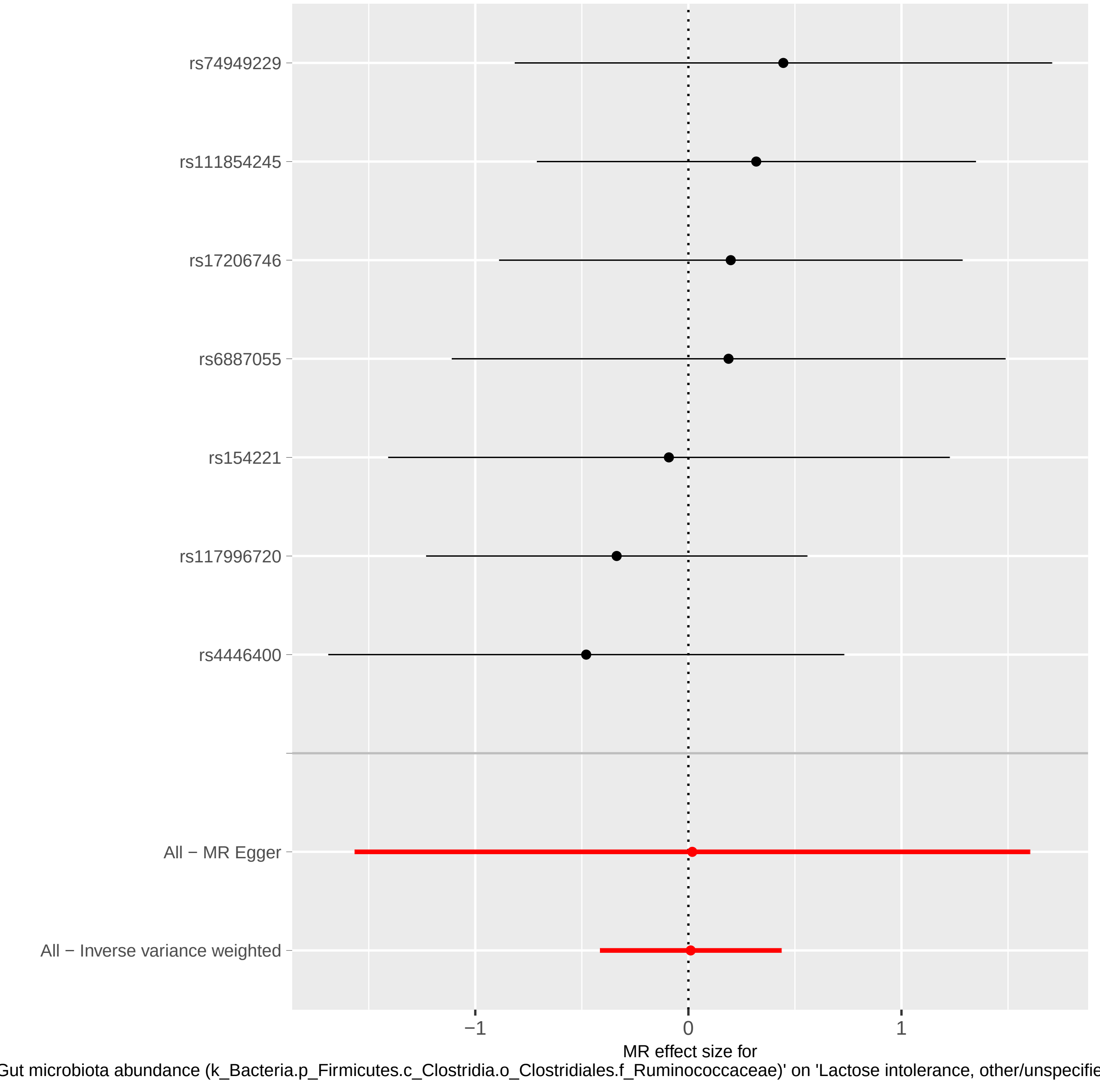
rs74949229
rs111854245
rs17206746
rs6887055
rs154221
rs117996720
rs4446400
All − MR Egger
All − Inverse variance weighted
−1
0
1
MR effect size for
Gut microbiota abundance (k_Bacteria.p_Firmicutes.c_Clostridia.o_Clostridiales.f_Ruminococcaceae)' on 'Lactose intolerance, other/unspecifie
